# A Critical Assessment of Generative Models for Synthetic Data Augmentation on Limited Pneumonia X-ray Data

**DOI:** 10.3390/bioengineering10121421

**Published:** 2023-12-14

**Authors:** Daniel Schaudt, Christian Späte, Reinhold von Schwerin, Manfred Reichert, Marianne von Schwerin, Meinrad Beer, Christopher Kloth

**Affiliations:** 1 Institute of Databases and Information Systems, Ulm University, James-Franck-Ring, 89081 Ulm, Germany; 2 DASU Transferzentrum für Digitalisierung, Analytics und Data Science Ulm, Olgastraße 94, 89073 Ulm, Germany; 3 Department of Computer Science, Ulm University of Applied Science, Albert–Einstein–Allee 55, 89081 Ulm, Germany; 4 Department of Radiology, University Hospital of Ulm, Albert–Einstein–Allee 23, 89081 Ulm, Germany

**Keywords:** deep learning, generative models, medical imaging, pneumonia, synthetic data

## Abstract

In medical imaging, deep learning models serve as invaluable tools for expediting diagnoses and aiding specialized medical professionals in making clinical decisions. However, effectively training deep learning models typically necessitates substantial quantities of high-quality data, a resource often lacking in numerous medical imaging scenarios. One way to overcome this deficiency is to artificially generate such images. Therefore, in this comparative study we train five generative models to artificially increase the amount of available data in such a scenario. This synthetic data approach is evaluated on a a downstream classification task, predicting four causes for pneumonia as well as healthy cases on 1082 chest X-ray images. Quantitative and medical assessments show that a Generative Adversarial Network (GAN)-based approach significantly outperforms more recent diffusion-based approaches on this limited dataset with better image quality and pathological plausibility. We show that better image quality surprisingly does not translate to improved classification performance by evaluating five different classification models and varying the amount of additional training data. Class-specific metrics like precision, recall, and F1-score show a substantial improvement by using synthetic images, emphasizing the data rebalancing effect of less frequent classes. However, overall performance does not improve for most models and configurations, except for a DreamBooth approach which shows a +0.52 improvement in overall accuracy. The large variance of performance impact in this study suggests a careful consideration of utilizing generative models for limited data scenarios, especially with an unexpected negative correlation between image quality and downstream classification improvement.

## 1. Introduction

The necessity for swift and dependable patient screening emerged as a key lesson from the COVID-19 pandemic. The development of machine learning models for aiding early pandemic clinical decisions is crucial, reducing diagnosis time and assisting emergency medical personnel [1]. However, a significant challenge in rapidly creating models for new infectious diseases is the limited access to high-quality data. This constraint is a common issue in the medical field, often stemming from privacy concerns [2] and high data acquisition costs. In radiology, all imaging modalities are affected equally (including X-rays, computed tomography, and magnetic resonance imaging), as well as various organ systems and diseases. Recently, as part of the COVID-19 pandemic, inflammatory changes in the lungs have come into focus, as these are of great importance in everyday life and have an impact on patients’ lives as well as hospital capacities [3]. Hence, it is imperative to effectively utilize machine learning models under scarce data conditions.

While methods like transfer learning and self-/semi-supervised learning exist, the performance of deep learning models is notably influenced by the data quantity, as shown theoretically [4,5] and empirically [6,7,8]. This study exemplifies such a scenario within the medical domain, focusing on a limited dataset. An analysis on chest X-ray (CXR) images pertaining to four different pneumonia causes is conducted, along with healthy patient images, with as few as 74 images for viral/non-COVID-19 cases. The objective is to leverage generative models to achieve reliable predictions despite the constraints of limited data. To date, using generative models for synthetic data augmentation on limited data is an under-explored research area. Although generative models are commonly used for larger datasets in medical imaging with a reported increase in performance [9,10,11,12,13], we do not see the same rigorous research towards scarce data scenarios, where such approaches would be most helpful. We aim to close this gap and initiate the discussion in this area.

**Figure 1:**
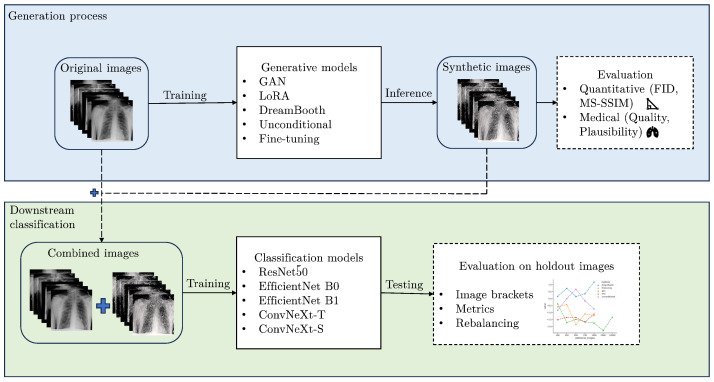
Schematic representation of the research problem of this work and the proposed evaluation framework.

This study provides a comprehensive evaluation of diffusion and Generative Adversarial Network (GAN)-based learning approaches, specifically aiming at improving performance of the downstream classification task of predicting COVID-19, other viral pneumonia, fungal pneumonia, bacterial pneumonia, and healthy cases on 1082 CXR images. We examine five different generative approaches and provide quantitative and medical assessments of image quality, diversity, and plausibility. The artificially generated images are used for synthetic data augmentation, where we measure the impact on performance for five different classification models. Additionally, varying amounts of synthetic images are added to the training data to further increase robustness of the evaluation. Although some generative approaches outperform our baseline models by a substantial amount, this study does not show an improvement in classification performance on average over all architectures and configurations. Despite that, class-specific metrics like precision, recall, and F1-score show a substantial improvement by using synthetic images, emphasizing the data rebalancing effect for the less frequent classes. This holds true when compared to a simple oversampling approach. Although we report better average classification improvement on this dataset in a previous study [14], this study deliberately does not utilize additional domain knowledge in the process, using only simple prompts for text-conditioned models and a non-domain specific text encoder instead. Figure 1 shows a schematic representation of our research approach. The code for this work can be found at: https://github.com/dschaudt42/synthetic_pneumonia (accessed on 30 November 2023). The 70,000 synthetic images produced in this work are available at: https://huggingface.co/datasets/dschaudt42/synthetic_pneumonia (accessed on 30 November 2023).

In summary, the main contributions of our work are:

- A comprehensive evaluation of diffusion and GAN-based learning approaches on a limited pneumonia X-ray dataset, testing the applicability of generative models in a scarce data scenario.
- Quantitative and medical assessments of image quality, diversity, and plausibility for synthetically generated images and show large gaps between the demonstrated generative approaches.
- Evaluation of synthetic data augmentation on a downstream classification task in a comprehensive manner, examining multiple classification architectures and additional image brackets for robust results. This study shows that higher quality images, as perceived by metrics and experts, do not necessarily lead to better classification performance. Furthermore, synthetic images can improve class-specific metrics substantially due to a data rebalancing effect, while aggregated performance metrics do not benefit in most cases.

## 2. Related Work

Generating high quality synthetic images is a field that gained a lot of traction with the inception of the GAN model [15]. In medical imaging, synthetic images have been used to translate between image modalities [16,17,18], enable sharing of privacy-protected data [19,20], and improve deep learning models on diverse downstream tasks [16,19,21,22,23].

Al Khalil et al. [24] propose a conditional GAN model, which increases segmentation performance on cardiac magnetic resonance images. The performance increase is especially noticeable when real and synthetic images are combined during training. Prasanna Das et al. [25] propose a conditional flow model to generate chest CT images and validate their approach by synthetic data augmentation for a downstream classification task of detecting COVID-19. Several GAN-based models have been proposed to improve performance on downstream COVID-19 tasks [9,10,11].

Recently, diffusion-based models have shown improved performances over GAN-based architectures in many domains [26,27,28]. He et al. [12] show that using synthetic data from large text-to-image models is a valid approach to downstream image recognition tasks, but do not consider smaller, more domain-specific datasets, like the ones presented in this work. Pinaya et al. [29] use latent diffusion models to generate synthetic brain MRI images, conditioned on the covariates age, sex, and brain structure volumes and compare them to inferior GAN-based baselines.

Chambon et al. [30] provide experiments on fine-tuning the different components of a Stable Diffusion model to secure domain-adaption for chest X-ray images. They found that the pre-trained variational autoencoder and the CLIP text encoder have a sufficient domain-adaption capabilities for chest X-ray images, and that fine-tuning the U-Net component is critical to improve image quality. Since we want to exclude further domain knowledge from our generation process, we also chose this approach of only fine-tuning the U-Net, while using simple, class-specific prompts. In a follow up work, the authors fine-tune the CLIP text encoder and report improved performance on a downstream classification task [23].

Müller-Franzes et al. [31] compare their latent denoising diffusion model, Medfusion, to GAN models on multiple medical imaging datasets of fundoscopy images, radiographs, and histopathology images. Packhäuser et al. [20] compare a PGGAN [32] model with a latent diffusion model on the ChestX-ray14 dataset [33] with a focus on privacy-enhancing sampling. Both found that classification performance does not increase when using the synthetic data, but found that the diffusion model generates higher quality images than the GAN model.

Most of the existing literature uses large amounts of samples to train generative models, even in the medical imaging domain with large image collections, sometimes with over 100,000 samples. Although there is undeniable evidence that generative models and deep learning models in general perform better with more samples [4,5,6,7,8], we argue that employing synthetic data is most useful in data scarce scenarios. If large datasets are available, the need for synthetic images can certainly be challenged. In this work, we try to test the limits of generative models in a data scarce scenario (n<50 for some classes), especially for downstream classification tasks.

## 3. Materials and Methods

We train and evaluate 5 different generative models on a limited pneumonia CXR dataset to improve downstream classification with synthetic data augmentation. This section describes the data, the generative models, and the training details for generative and classification models.

### 3.1. Data

**Figure A1:**
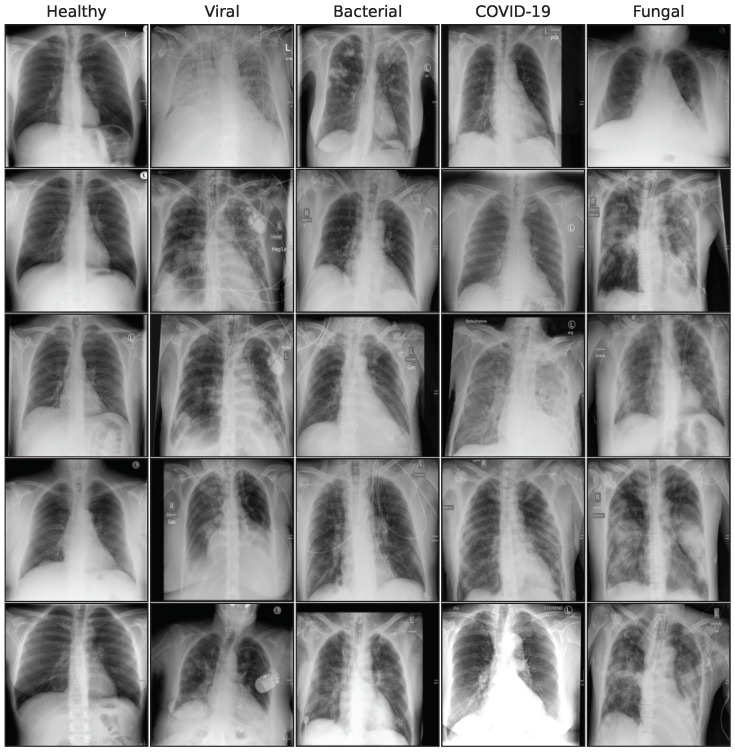
Sample images from original dataset.

The dataset of this study was initially described in Schaudt et al. [14] and contains 1082 chest X-ray images from a total of 828 patients (342 female and 486 male) with ages ranging from 18 to 89 years (mean age 52.52 ± 17.45 years). Radiographs were acquired during chest radiography examinations due to clinical symptoms on a portable flat detector (Flurospot Compact Siemens Healthcare, Erlangen Germany and DRX Evolution Carestream, Stuttgart, Germany). The ethics board of the Medical Faculty and the University Hospital in Ulm approved this retrospective data evaluation study and waived the informed consent requirement (No. 271/20). All methods were carried out in accordance with relevant guidelines and regulations. Figure A1 in the Appendix A shows a sample collection of 5 images per class.

#### 3.1.1. Data Acquisition

Radiographs were retrieved through retrospective analysis of the local radiology department database. Bacterial infections were ascertained via sample material collected from bronchoalveolar lavage or sputum, while fungal infections were confirmed through positive microscopy or culture. Diagnosis of COVID-19 in all patients was established using nasopharyngeal swabs, followed by RT-PCR assay. Virus detection and verification were performed on bronchoalveolar lavage samples using a commercially available real-time PCR assay.

#### 3.1.2. Data Labeling

Images were differentiated and labeled by a dedicated thoracic radiologist (CK) with 9 years of experience in lung imaging with the following distribution: 673 (62.20%) healthy patients (H), 125 (11.56%) fungal infection (F), 110 (10.17%) COVID-19 (C), 100 (9.24%) bacterial infection (B), and 74 (6.84%) other viral infection (V). Table 1 shows the demographic variables for training and validation cohorts used in this study.

#### 3.1.3. Holdout Splits

To assess our models, a random subject-based holdout method is employed, ensuring patient non-overlap between splits. Approximately 20% of the images are designated as validation data, while maintaining label distribution as evenly as possible within this constraint. Table 2 shows the exact label distribution for training and validation splits. Prolonged training times for generative models prohibited the use of cross-validation as an evaluation strategy.

### 3.2. Image Synthesis

**Figure A2:**
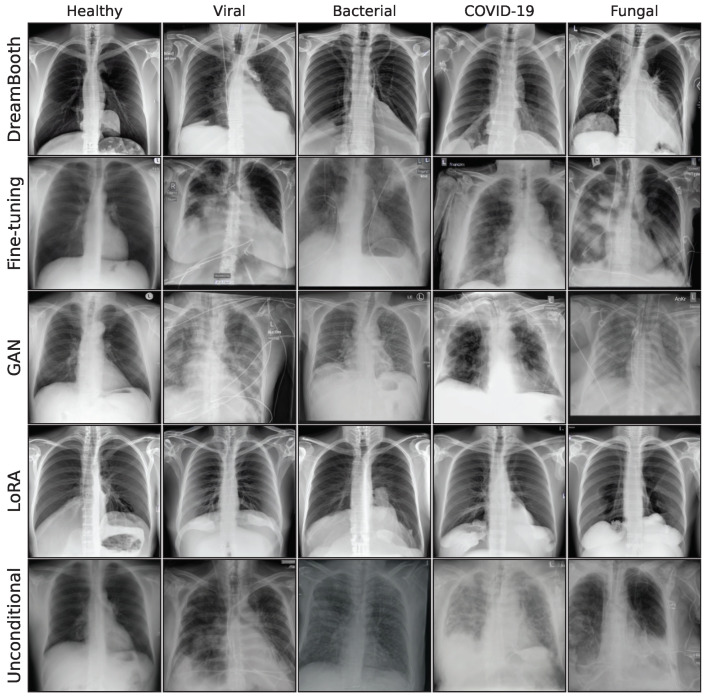
Sample synthetic images from all classes and methods.

This subsection describes the generative models used in this work. We utilize a special GAN model, a Denoising Diffusion Probabilistic Model (DDPM), and 3 different fine-tuning approaches for a Stable Diffusion [34] model: standard fine-tuning, Low-Rank Adaption (LoRA), and DreamBooth. Our aim is to compare the performance of a GAN model to more recent diffusion-based architectures, building on the GAN proposed in Schaudt et al. [35]. Figure A2 in the Appendix A shows a collection of synthetic images for all generative models.

#### 3.2.1. GAN

**Figure A3:**
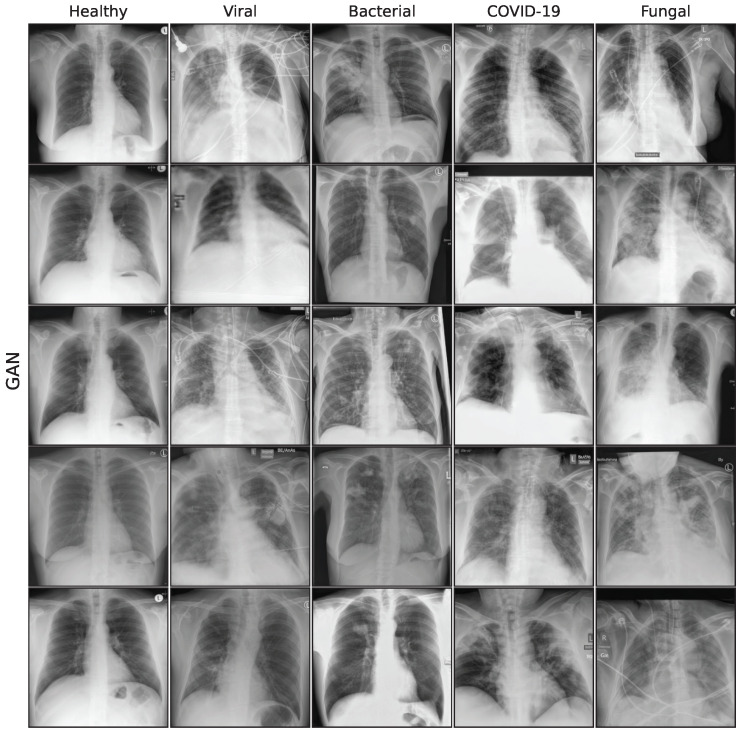
Sample synthetic images from GAN model for all classes.

Our GAN [15] model is based on the StyleGAN architecture [36] and uses the WGAN-GP-loss and Adam optimizer [37] as in Karras et al. [36]. To ensure a stable training process on our limited study data, differentiable augmentation, as introduced by Zhao et al. [38], is employed. In our internal testing this is a critical step to achieve high quality images from limited data, since the primary source of training instability is the discriminator memorizing the training data. To mitigate this, both real and generated images are augmented with differentiable operations before being fed to the discriminator, facilitating generator training through backpropagation. During training, the resolution increases progressively to stabilize training and achieve higher resolution images. Furthermore, we find that the original StylGAN worked very well with the differentiable augmentation approach. More recent GAN architectures showed increased training complexity with subpar results in our testing. Figure A3 in the Appendix A shows a collection of synthetic images for the GAN model.

#### 3.2.2. Unconditional

**Figure A4:**
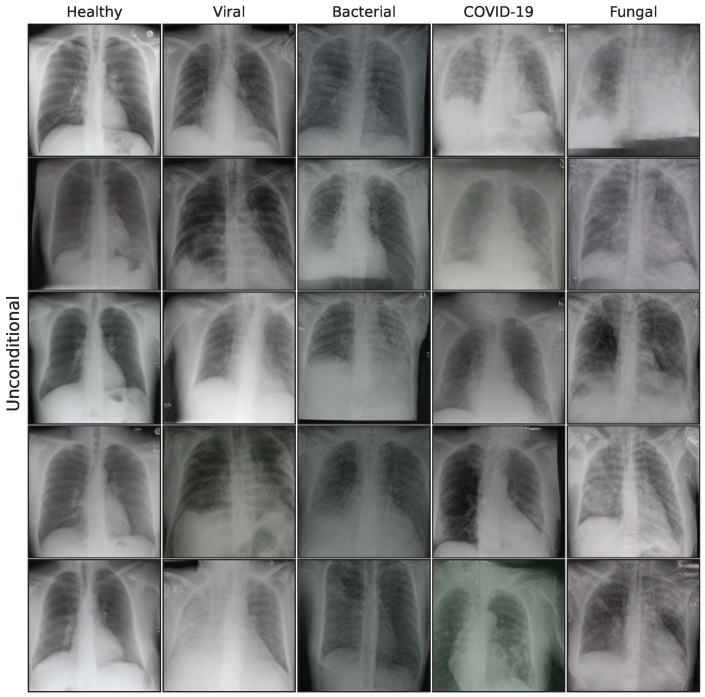
Sample synthetic images from unconditional DDPM model for all classes.

The unconditional diffusion model employs a DDPM scheduler [39] in conjunction with a U-Net model [40]. DDPM applies forward and backward diffusion processes, while backward diffusion applies Gaussian noise to an image in a scheduled manner, forward diffusion denoises the image again using a predictive model. The predictive model in this case is a U-Net, which predicts the noise residual on the image. Both processes are executed for a finite number of time steps *T*, starting with t=0 sampling a real image from the training data distribution. For sufficiently large *T* the forward diffusion process produces an isotropic Gaussian distribution at t=T via a gradual process. Reversing this diffusion process enables the generation of new images from pure noise. Figure A4 in the Appendix A shows a collection of synthetic images for the unconditional model.

#### 3.2.3. Fine-Tuning

**Figure A5:**
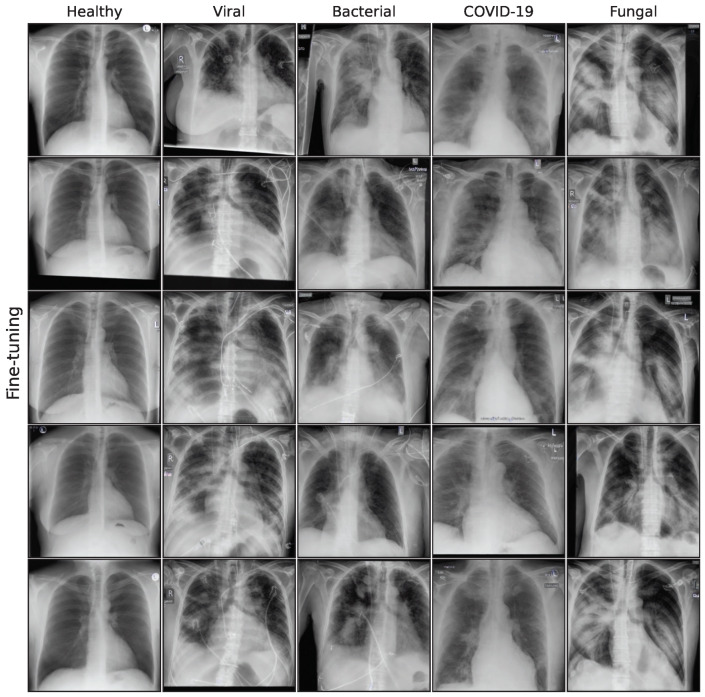
Sample synthetic images from fine-tuning model for all classes.

A standard fine-tuning regime for Stable Diffusion is used, which is a specific type of diffusion model for text-to-image applications [34]. Compared to regular diffusion models, Stable Diffusion operates in latent space, and utilizes a text encoder to condition image generation on text inputs. Images are compressed to a latent representation via an autoencoder component to reduce dimensionality and enable faster training. As in Rombach et al. [34], a frozen, pre-trained text encoder of a CLIP model [41] is used for the text embeddings. The denoising process is performed by a U-Net [40], which is being fine-tuned. Figure A5 in the Appendix A shows a collection of synthetic images for the fine-tuning model.

#### 3.2.4. LoRA

**Figure A6:**
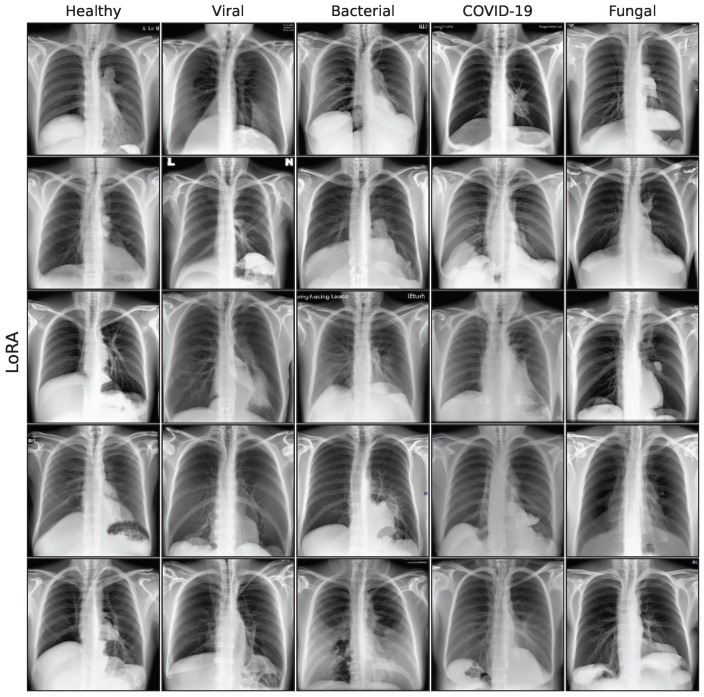
Sample synthetic images from LoRA model for all classes.

Low-Rank Adaption of Large Language Models (LoRA) [42] is a training technique, that was originally proposed to efficiently fine-tune large language models. It freezes pre-trained model weights and adds trainable layers in transformer blocks, reducing trainable parameters substantially. In this work, LoRA is applied to fine-tune a Stable Diffusion model [34], by applying the LoRA weights (rank-decomposition matrices) to the cross-attention layers that relate the image representations with the prompts that describe them. This makes the training process fast and reduces compute requirements, as well as model size. Figure A6 in the Appendix A shows a collection of synthetic images for the LoRA model.

#### 3.2.5. DreamBooth

**Figure A7:**
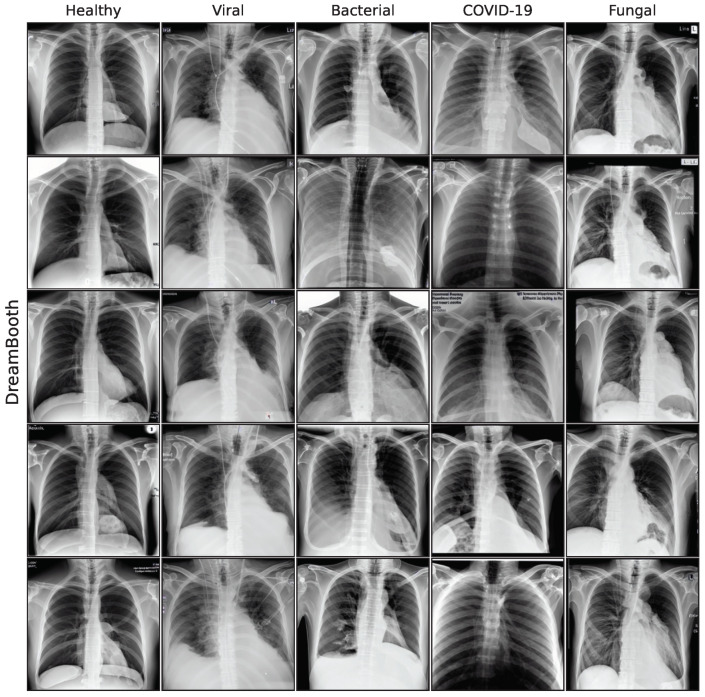
Sample synthetic images from DreamBooth model for all classes.

DreamBooth [43] is a specified method to personalize text-to-image diffusion models with new subjects in a few-shot manner. Similar to standard fine-tuning, the approach fine-tunes the U-Net component on domain-specific images, while keeping the autoencoder and text encoder frozen. To prevent catastrophic forgetting and adapt to new concepts, a prior-preserving loss is used, which pairs images and prompts from the prior [43]. This technique enables the generation of high-fidelity CXR images with simple pathologies through text conditioning. However, overfitting can still occur, and image diversity remains limited. Therefore, the number of training iterations are limited. Figure A7 in the Appendix A shows a collection of synthetic images for the DreamBooth model.

### 3.3. Training Details

This subsection contains all training configurations and hyperparameter settings for generative and classification models. Since we want to evaluate the model performance without the integration of further domain knowledge, we use simple prompts for our text-conditioning models and keep the text encoder weights frozen. PyTorch v1.13.1 [44] is used to carry out the computations.

#### 3.3.1. Generative Models

The GAN model implementation is based on Zhao et al. [45] and Seonghyeon [46]. All diffusion model implementations are based on Hugging Face [47], especially the diffusers library [48] in version 0.17.1. The Stable Diffusion weights were obtained from the CompVis/stable-diffusion-v1-4 repository. For training and inference of the text-conditioned models LoRA, DreamBooth, and fine-tuning, we used the following prompts: “An X-ray image of the lung with {viral, bacterial, COVID-19, fungal} pneumonia” or in the case of a healthy patient: “An X-ray image of the lung, healthy patient, no signs of pneumonia”. These rather simple prompts were chosen for a clear distinction between the classes without integrating further domain specific knowledge. We also expect that providing more image-specific prompts might not be beneficial for such a small dataset, where detailed descriptions will most likely not repeat. Throughout the following sections, unless noted otherwise, the architectures remain unaltered, with the exception of disabling the built-in “safety checker” due to its high false-positive rate with medical prompts. Table 3 shows the hyperparameter configuration for all generative models. The configurations mostly follow the default implementation, with some adaptions considering our hardware. Note that we do not employ excessive hyperparameter optimization, due to infeasible training times. All models were trained in a multi-GPU setting with two NVIDIA RTX 3090. The maximum training steps are varied slightly to account for differences in training and inference times between models. Since the GAN model can generate images much more quickly, we increased the training iterations. The total computation time is similar for all models, except DreamBooth, which was fine-tuned for only 1500 iterations due to sharp decrease in image quality.

#### 3.3.2. Classification Models

We demonstrate the effect of synthetic data augmentation on the same study data as in Schaudt et al. [14] in a downstream classification task. To validate our approach, we train multiple model architectures with this process: ResNet50 [49], EfficientNet-B0 [50], EfficientNet-B1 [50], ConvNeXt-T [51], and ConvNeXt-S [51]. A broader selection of older and newer state-of-the-art models were chosen, which have been used extensively in academic literature. All experiments were repeated 5 times to increase the robustness of our results. Baseline models were trained for all architectures without synthetic data augmentation as a point of reference. All models were pre-trained on ImageNet [52], providing well-calibrated initial weights. Unlike traditional transfer learning, we update all gradients to account for shifts in image distribution. ImageNet’s diverse dataset differs significantly from our desaturated CXR data. The final layer was replaced with a linear layer featuring 5 output nodes, one for each class.

We employ an augmentation pipeline for all classification models to increase image variations and reduce overfitting during model training, which is common for many image domains [53,54,55]. This pipeline was inspired by the winning solution to the 2021 SIIM-FISABIO-RSNA Machine Learning COVID-19 Challenge [56] and is shown in Table 4. Augmentations are carried out by the Albumentations library [57]. All classification models use an Adam [37] optimizer with $\beta_{1},\beta_{2}=0.9,0.999$ momentum and cross-entropy loss with a batchsize of 8. The learning rate is initialized at $1\times 10^{-4}$ and follows a cosine annealing function. All models are trained for 60 epochs. To alleviate overfitting, a dropout layer was added before the classification layer with p=0.5.

## 4. Results

In this section, we examine the results of the five generative methods presented in the following categories: generative performance, medical assessment, and classification performance. In generative performance, we look at the performance metrics FID [58] and MS-SSIM [59] to quantify the fidelity and diversity of the generated images. In medical assessment, a dedicated thoracic radiologist (C.K.) with 9 years of experience in lung imaging assessed the quality and plausibility of the generated images from a medical perspective. In classification performance, the effect of synthetic data augmentation on a downstream classification task is evaluated.

### 4.1. Generative Performance

Generated images should be similar to the underlying, real image distribution (fidelity), and ideally show a large variability in possible outcomes (diversity). Fidelity and diversity can be measured by the Fréchet Inception Distance (FID) [58] and the Multi-Scale Structural Similarity Index (MS-SSIM) [59], respectively. Both metrics are commonly used in generative image tasks. We calculate the FID based on the final 2048 feature layer of a pre-trained Inception V3 [60] model, as is standard. The distance is calculated by comparing 50 synthetic images of each class to 50 real images. The MS-SSIM (Gaussian kernel size 11; sigma, 1.5), which is a generalization of the SSIM [61], is calculated by a pairwise comparison of all combinations of the same 50 synthetic images and taking the average. Lower values of FID and MS-SSIM show larger fidelity and diversity. It should be noted that the metrics used are contingent on the reference samples and implementation, making direct comparisons with other studies challenging [62].

**Figure 2:**
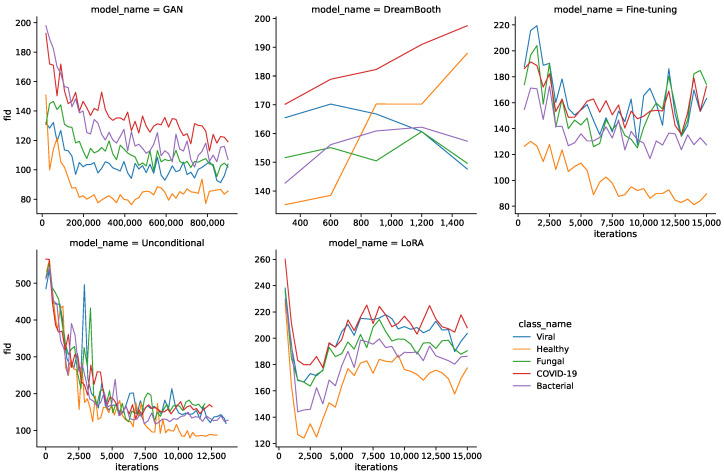
FID curves during training for all models.

Figure 2 shows the FID values during the training for all models and classes. Interestingly, FID values do not simply decrease during training for all models, but can also increase towards the middle or end of the training process. This is especially apparent for DreamBooth, LoRA, and fine-tuning models, where the FID increases from the start or later during training. The LoRA model sees a sharp drop in FID values in the beginning and shows another decline towards the end of training.

**Figure 3:**
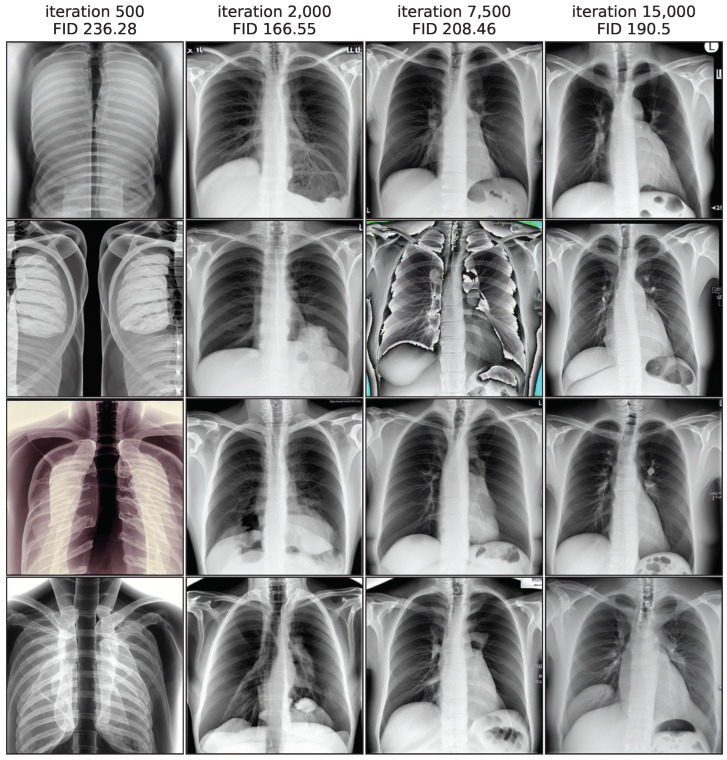
Non-monotonic FID progression for LoRA model, showing 4 random samples per iteration.

Figure 3 illustrates this by showing image samples of the LoRA model from different training iterations, while images from iteration 500 exhibit the largest FID values and a low quality, the quality improves substantially in iteration 2000. In iteration 7500 the FID rises again and the quality decreases, as shown clearly by a faulty image. In the end of the training process, the FID decreases again slightly and quality seems to increase, while not fully reaching earlier levels. This non-monotonic FID progression confirms the general usefulness of monitoring the FID values during the training process to pick the best model iteration.

**Figure 4:**
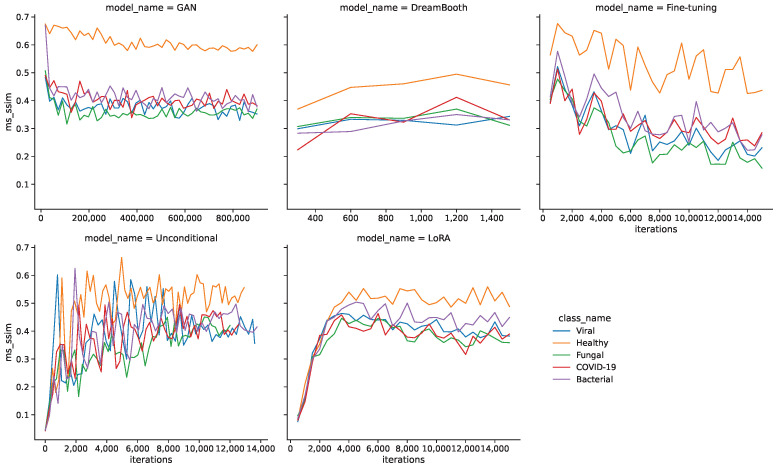
MS-SSIM curves during training for all models.

Another noteworthy observation is a difference in FID curves between classes. Healthy images generally have lower FID values than the pathological classes, which might stem from a substantial difference in quantity of the underlying real images. Figure 4 shows the MS-SSIM values during the training for all models and classes. In this case, the larger quantity of healthy images seems to be a disadvantage, as they generally produce higher MS-SSIM values and therefore show a lower diversity.

Table 5 shows the resulting minimum FID and MS-SSIM values for all models and classes. The GAN model shows the lowest FID values for all classes by a sizable margin, followed by unconditional and fine-tuning models. DreamBooth and LoRA models generally show larger FID values and therefore lower fidelity images. The MS-SSIM values are mostly similar for the minimum FID iterations, with unconditional and DreamBooth models having slightly higher values.

### 4.2. Medical Assessment

Quantitative distance metrics can be a good first indicator of image quality, but they do not provide any medical assessment of image quality or pathological plausibility of the synthetic images. We argue that an evaluation by a human expert is critical in such a sensitive medical setting. A dedicated thoracic radiologist (C.K.) with 9 years of experience in lung imaging has therefore assessed the quality and plausibility of the generated images from a medical perspective.

The quality of an image was assessed on a scale of 1 (lowest quality) to 5 (highest quality). Important aspects of the quality assessment are that thorax and lungs are shown as a whole, so that the anatomy is reproduced correctly and to scale. The sharpness and contours must be reproduced correctly. If a pathology was present, it was evaluated according to its characteristic appearance on a scale from 1 to 3 (1 = completely inappropriate, 2 = partly characteristic, 3 = characteristic). Important aspects of the plausibility assessment are to what extent the typical appearance of pneumonia is reproduced. This includes the density, the sharpness compared to the lungs, the relationship to other anatomical structures (heart, pleura) as well as the distribution pattern within both lungs (centrally emphasized, peripherally emphasized, division into the individual lung lobes).

**Figure 5:**
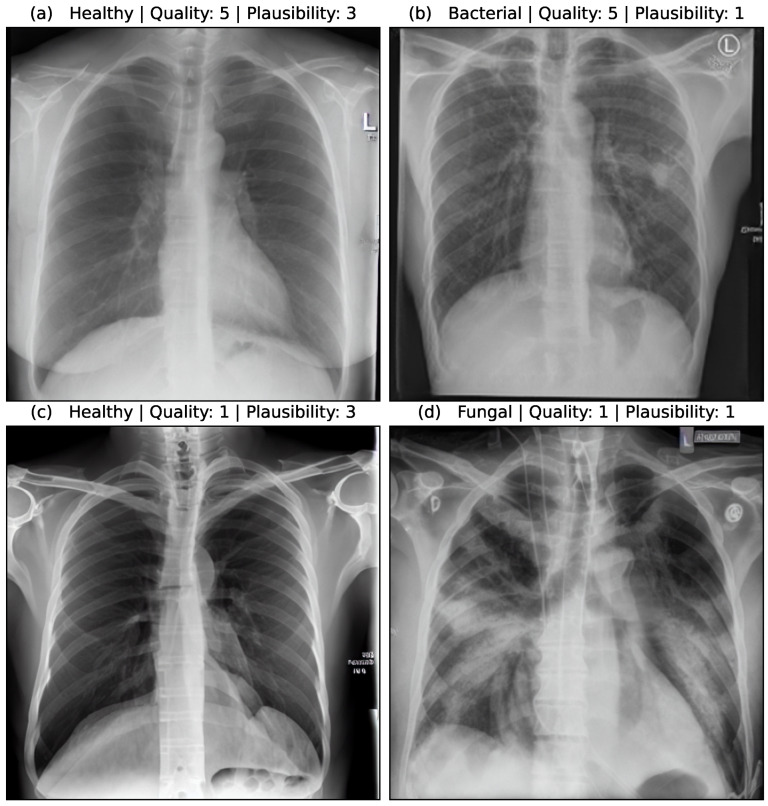
Medical assessment of quality and plausibility for 4 synthetic image samples. (**a**) Healthy case with high quality and plausibility. (**b**) Bacterial case with high quality but low plausibility. (**c**) Healthy case with low quality but high plausibility. (**d**) Fungal case with low quality and plausibility.

Figure 5 shows the assessment of four synthetic image samples with high/low quality and high/low plausibility scores. In (a, b) the anatomical structures are reproduced realistically and the proportions are accurate. The pleura, diaphragm, heart contour and hilar vessels are reproduced with absolute precision. The breast shadow is also reproduced exactly in (a), which simulates a woman as the gender of the patient. The quality rating is accordingly rated score 5 without any gradations. The healthy state in image (a) is shown regularly with a quality score of 5. The bacterial pneumonia in (b) is rather atypical, a suspect bronchial carcinoma from the simulated image is more realistic. It does not reflect peripheral inflammation in the sense of bronchopneumonia or lobar pneumonia, hence the assessment of plausibility as inappropriate (score 1). Image (c) appears artificial in appearance, the diaphragm contours, the heart silhouette and the bones are not realistically reproduced, the image quality is only rated with score 1. In contrast, no pathology of the lung parenchyma is recognizable, but this is still realistic and therefore rated as score 3 in terms of plausibility. Image (d) also appears artificial in appearance, in particular the hints of foreign material/lines and heart contours are unrealistic (quality score 1). The inflammations of the lungs described also seem unrealistic for any type of pneumonia; a fungal infection is unrealistically shown (plausibility score 1).

**Figure 6:**
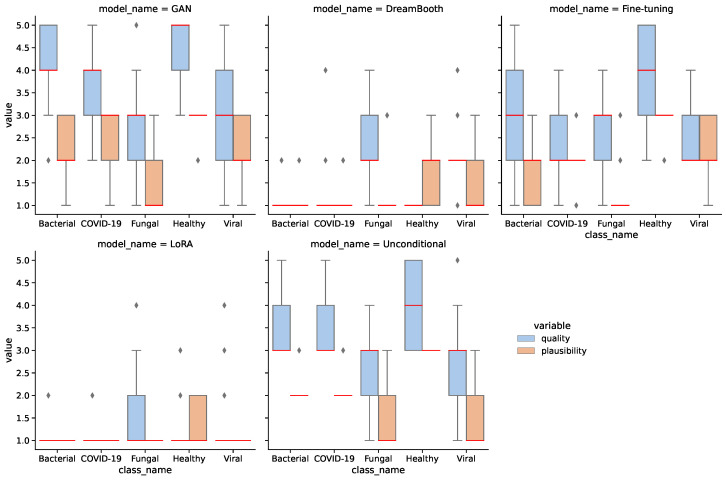
Medical assessment of quality and plausibility for all models. Red line shows median values.

We assessed 25 synthetic images of each class for each model in quality and plausibility, totaling 625 images. Figure 6 shows the results of the assessment for all models and classes. A Kruskal–Wallis test [63] confirms that the difference between models for both quality and plausibility is significant with p<0.0001. A Dunn–Bonferroni post hoc test [64] on quality shows significant differences (p<0.05) between most models, except for the GAN-unconditional pair (p=0.713), the unconditional-fine-tuning pair (p=0.51), and the DreamBooth-LoRA pair (p=0.3).

The assessment confirms that the GAN model generates the highest quality and most plausible images, followed by the unconditional and fine-tuning models. DreamBooth and LoRA models seem significantly weaker than the other models. Additionally, the healthy images have higher quality and plausibility on average than the other classes. This is mostly due to larger image quantity and reiterates on the merit of larger data for generative models. Table 6 aggregates the assessment results for all models. It should be noted that the medical assessment was not used to filter images based on quality for the downstream classification task. Although this might affect the classification task, we deliberately want to omit the integration of further domain knowledge to obtain an unbiased estimation of downstream performance. Furthermore, a comprehensive medical assessment of 70,000 synthetic images would be infeasible.

### 4.3. Classification Performance

The effect of using the generated images as synthetic data augmentation to improve a classification downstream task is examined, utilizing synthetic images from the lowest FID iterations for all generative models. A selection of these final images is shown in the Appendix A. The performance is measured on multiple model architectures: ResNet50 [49], EfficientNet-B0 [50], EfficientNet-B1 [50], ConvNeXt-T [51], and ConvNeXt-S [51]. The respective baseline models do not use additional synthetic images for training and their performance has been reported in Schaudt et al. [14]. To gain a better understanding on the synthetic data augmentation methodology, five different generative models with five different classification architectures are benchmarked and also vary the amount of additional synthetic images. All classification model trainings have been repeated 5 times with a different seed to obtain a robust and comprehensive outlook on the expected performance gain. Since the GAN model can generate images much more quickly than the diffusion-based models (in about 1/10th time), we want to examine this advantage by adding even more synthetic images into the training. The +5000 and +10,000 image brackets are therefore only evaluated for the GAN model, since generating such large quantities would be unfeasible for the other models in our setting.

Table 7 reports the accuracy for the presented classification models, trained with additional synthetic images from the presented generative models. The best model is ConvNeXt-S, trained with 250 additional synthetic images from the unconditional model with 81.11% accuracy. This is a notable increase of +2.58 percentage points from the baseline variant, while the best performing models for each architecture all use synthetic images, the results show a large variation in performance across all methods and image brackets. Many configurations even see a decline in classification performance.

Table 8 shows aggregated results as the average change in accuracy in percentage points for all models from baseline over all additional image brackets. The only generative model with a positive impact on classification performance over all classification models is DreamBooth with an average improvement of 0.52. Interestingly, specific generative models can have a substantially higher than average impact on performance for specific classification models. This suggests that some model combinations fit very well together, while others do not. The classification model that shows the highest improvement on average across all generative models is ConvNeXt-S with an increase of 0.12 over baseline.

**Figure 7:**
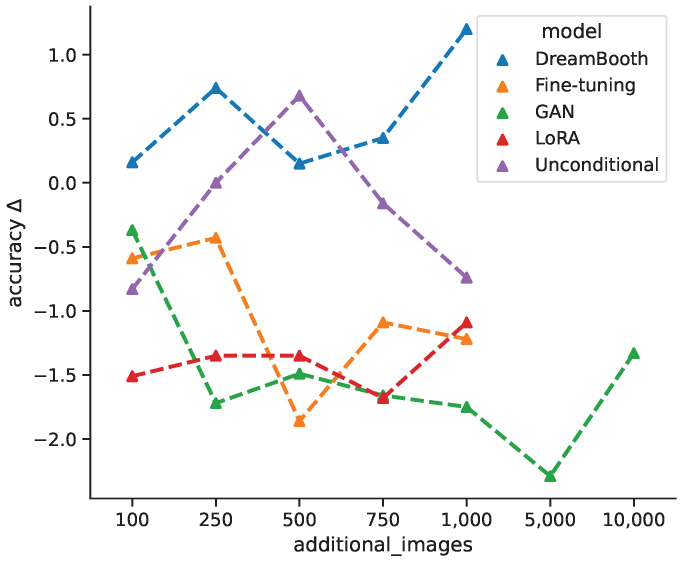
Average change in classification accuracy for all models from baseline per additional images bracket.

Figure 7 shows the average change in classification accuracy for all generative models from baseline per additional image bracket over all classification models, while DreamBooth favors larger quantities of synthetic images, the unconditional model shows an improvement for intermediate quantities, which decreases again for +750 and +1000 images. Both fine-tuning and GAN models show decreased performances for larger quantities, with a slight incline towards the maximum quantities. This could suggest that even more images might have a positive impact. The LoRA models are largely invariant under different image quantities. Varying the amount of additional synthetic images for training does not follow a clear pattern and seems to depend largely on the specific generative model. For cases where classification accuracy first decreases and then increases could be due to the classification model shifting from learning original features towards generated features. In general, the change in classification accuracy between different models could result from the models ability to generate classifiable features.

To examine class specific performances, we focus on the classification model with the best overall accuracy, which is ConvNeXt-S. Table 9 shows the precision, recall, and F1-score for all generative models for each class. The metrics have been calculated based on the best performing image bracket for each generative model. We also include a ConvNeXt-S model trained with randomly oversampled classes as a simpler approach to rebalance class distribution during training. Generative approaches show higher recall values for most classes, with sizable differences for some classes (LoRA 0.36 vs. baseline 0.1 for viral cases). All pathological classes show substantially higher recall and F1 values for generative approaches, especially for bacterial, fungal, and viral cases. The baseline model shows the best precision and F1 values for healthy cases, as well as the best precision for COVID-19. This is not very surprising, since these classes are the most frequent and do not benefit as well from the rebalancing effect of synthetic data augmentation. Despite the lower accuracy of the generative approaches, class-specific performances can suggest the use of generative models in imbalanced learning scenarios. This holds true when compared to a simple oversampling approach, which does perform worse for most classes and completely misses the bacterial cases.

## 5. Discussion

Surprisingly, we found that higher image quality does not translate to better performance on a downstream classification task. Looking at the accuracy, most models performed worse with synthetic data augmentation, which is in line with Müller-Franzes et al. and Packhäuser et al. [20,31]. Only the DreamBooth model leads to an overall improvement across all examined classification architectures. We can only guess why image quality does not translate to improved classification models. Images from DreamBooth and LoRA models seem to exhibit higher contrast and unrealistic visibility of the bone structure and appear more cartoon-like in general. This could lead to an indirect regularization effect, leading models to broader areas of the loss function and reduce overfitting. Additionally, those models are pre-trained and already had exposure to limited amounts of chest X-ray images, which could potentially increase image diversity.

It could also be the case that our quality and plausibility assessment does not accurately evaluate whether the generated images hold features that are relevant to classification models. We think that the appearance of typical pathological patterns should, in theory, be the decisive feature used by classification models. Due to the black box nature of these models, it is not possible to finally conclude which features are being used for classification. Even attribution methods like GradCAM [65] do not map pixel attributions to features in a meaningful way or aggregate the information over many images. Although there might be other metrics for a medical assessment that correlate better with classification performance, we think that the chosen ones are meaningful from the perspective of a human evaluator and present the medical perspective.

Some models benefited more from synthetic data augmentation than others. For example, ConvNeXt-S shows an average improvement of 0.12 percentage points, while EfficientNet-B0 shows a decrease in accuracy of −2.09 percentage points on average. Since EfficientNet-B0 and EfficientNet-B1 show very different improvements (−2.09 pp. vs. 0.02 pp.), model size alone is not a decisive factor to predict model improvement from synthetic data augmentation. More recent architectures with higher capacity show larger improvements on average. We also examined the effect of adding different amounts of synthetic images to the training data. The results are inconclusive with some models benefiting from more images (DreamBooth and LoRA), while others show a performance decline (GAN and fine-tuning). Since the behavior seems to be model-specific, we suggest to experiment with different settings, especially when inference times are short and new images can be generated quickly.

Class-specific metrics like precision, recall, and F1-score, have shown that synthetic data augmentation beats the baseline performance on most classes. This is especially true for classes with low frequency like viral and fungal pneumonia. In cases of imbalanced learning problems, synthetic data augmentation to rebalance class distributions provide a meaningful benefit. This technique performs better than a simple oversampling approach in this study, but comparisons to other sampling methods [66,67,68] might be an interesting direction for further research. Although we could not confirm an overall improvement for a downstream classification task in this study, we did not cherry-pick model configurations where generative models exhibit sizable improvements over baseline models, but opted to give a comprehensive and robust outlook on the expected performance increase over many different scenarios instead. Furthermore, the usefulness of synthetic data exceeds the synthetic data augmentation approach, for example by using synthetic samples from a different institution for pre-training as shown in [69] for 3D medical images.

Distinguishing between different pneumonia types has advantageous clinical implications, since they require different treatment regimes. Early indications of the cause can help facilitate effective drug treatments, for example in the use of antibiotics or antimicrobial drugs. If the pneumonia is caused by an infectious disease like COVID-19, controlling virus spread becomes an important task and early evidence of such diseases can be very helpful. Early diagnosis and appropriate treatment are essential to prevent complications and improve outcomes. In cases of immunosuppression, e.g., after bone marrow transplantation, a precise differentiation between different infections can be challenging and of crucial importance for the patient. A fast and correct identification is therefore necessary for the survival of the patient. The use of AI can help to break down and identify the correct infection which can often be very similar in appearance [70].

Our work has limitations. We did not fine-tune the text encoder component of our Stable Diffusion models, which could lead to improved image quality, although recent literature seems to be indecisive on this effect [23,30]. We also chose rather simple prompts for text-conditioning of our Stable Diffusion models. Although more detailed prompts could lead to better results, we deliberately wanted to measure the effect of synthetic data augmentation without including further domain knowledge. We have already shown that incorporating domain knowledge can help to improve classification models on this study data [14]. This also applies to filtering generated images for quality and plausibility before using them on the downstream classification task. Since human evaluation is infeasible for large quantities of images, employing another classification model to filter out bad images could be a promising future approach.

## 6. Conclusions

In this work, five different generative models for a small pneumonia chest X-ray dataset were evaluated, giving a quantitative and medical assessment of image quality and pathological plausibility. Furthermore, the usefulness of these models as part of a synthetic data augmentation on a downstream classification task was examined. We compare a GAN-based model with diffusion and latent diffusion models on five different classification model architectures to obtain a comprehensive overview of the expected performance gain for synthetic data augmentation. Images generated by the GAN model have shown the best quality in both quantitative and medical assessment and are most plausible as they outperform more recent architectures. Unconditional DDPM and fine-tuning of a Stable Diffusion model follow closely, while the recent fine-tuning approaches LoRA and DreamBooth did not achieve satisfying results in this study.

Interestingly, synthetic healthy findings often exhibit higher quality than pathological findings. Aside from being the most frequent class in this study, we assume that pathological patterns increase image complexity and vary far more than a normal healthy state. We found that the quantitative assessment based on the FID score leads to the same ranking in image quality than our medical assessment. Therefore, we rate the FID score as a solid measure for image quality. Furthermore, image quality and medical plausibility are closely correlated. We have shown that FID scores can fluctuate significantly during training for generative models and therefore advocate to track these metrics closely to decide for the best model checkpoints. In conclusion, this study gives a realistic estimation on the expected performance gain of synthetic data augmentation in a scarce data scenario and encourages many possible directions for further research.

## Figures and Tables

**Table 1:** Summary of demographic variables and imaging protocol variables of CXR data for training and validation cohorts used in this study. Age and sex statistics are expressed on a patient level, while imaging view statistics are expressed on an image level with anteriorposterior (AP) and posterioranterior (PA) views. Reprinted from Schaudt et al. [14].

Variable	Group	Training Data	Validation Data
Age	mean ± std	51.75 ± 17.54	53.82 ± 18.05
	<20	20 (2.31%)	3 (1.38%)
	20–29	110 (12.72%)	29 (13.36%)
	30–39	105 (12.14%)	22 (10.14%)
	40–49	127 (14.68%)	28 (12.90%)
	50–59	191 (22.08%)	58 (26.73%)
	60–69	171 (19.77%)	26 (11.98%)
	70–79	102 (11.79%)	36 (16.59%)
	80–89	39 (4.51%)	15 (6.91%)
Sex			
	male	533 (61.62%)	134 (61.75%)
	female	332 (38.38%)	83 (38.25%)
Imaging view			
	AP	238 (27.51%)	68 (31.34%)
	PA	625 (72.25%)	149 (68.66%)

**Table 2:** Absolute and relative sample distribution for training and validation splits. Reprinted from Schaudt et al. [14].

Label	Training Data	Validation Data
Healthy	543 (62.77%)	130 (59.91%)
Fungal infection	96 (11.1%)	29 (13.36%)
COVID-19	87 (10.06%)	23 (10.60%)
Bacterial infection	81 (9.36%)	19 (8.76%)
Viral infection	58 (6.71%)	16 (7.37%)

**Table 3:** Training settings for all generative models. GAN batchsize and learning rate is provided in ranges and depends on the resolution of the progressive growing process.

Hyperparameter	Fine-Tuning	Unconditional	LoRA	DreamBooth	GAN
optimizer	Adam [37]	Adam	Adam	Adam	Adam
loss function	MSE	MSE	MSE	MSE	WGAN-GP
batchsize	1	8	1	1	[16,32]
learning rate	$1\times 10^{-5}$	$1\times 10^{-4}$	$1\times 10^{-5}$	$1\times 10^{-6}$	[$5\times 10^{-4}$,$1\times 10^{-3}$]
learning rate scheduler	constant	cosine	constant	constant	constant *
max training steps	15,000	13,750	15,000	1500	37,500
optimizer momentum $\beta_{1},\beta_{2}$	0.9, 0.999	0.95, 0.999	0.9, 0.999	0.9, 0.999	0.0, 0.99
optimizer epsilon	$1\times 10^{-8}$	$1\times 10^{-8}$	$1\times 10^{-8}$	$1\times 10^{-8}$	$1\times 10^{-8}$
weight decay	$1\times 10^{-2}$	$1\times 10^{-6}$	$1\times 10^{-2}$	$1\times 10^{-2}$	0.0
mixed precision	fp16	no	bf16	bf16	no
EMA	yes	no	no	no	no
resolution	512	256	512	512	256
gradient accumulation steps	4	1	4	4	-
gradient checkpointing	yes	no	yes	yes	no
max gradient norm	1.0	1.0	1.0	1.0	-
lr warmup steps	0	500	0	0	0
denoising steps	50	250	50	50	-
LoRA scale	-	-	1.0	-	-

* GAN learning rate is constant for each resolution step in the progressive growing process.

**Table 4:** Augmentation pipeline for classification models. Augmentations are carried out with Albumentations library [57].

Augmentation	Parameters	Probability
Resize	height=224, width=224	1.0
ShiftScaleRotate	scale_limit=0.5, rotate_limit=10, shift_limit=0.1	1.0
One of:		0.9
[CLAHE,	clip_limit=4.0, grid_size=(8,8)	1.0
RandomBrightnessContrast,	brightness_limit=0.2, contrast_limit=0.2, brightness_by_max=True	1.0
RandomGamma]	gamma_limit=(80,120)	1.0
One of:		0.9
[Sharpen,	alpha=(0.2,0.5), lightness=(0.5,1.0)	1.0
Blur,	blur_limit=7	1.0
MotionBlur]	blur_limit=7	1.0
One of:		0.9
[RandomBrightnessContrast,	brightness_limit=0.2, contrast_limit=0.2, brightness_by_max=True	1.0
HueSaturationValue]	hue_shift_limit=20, sat_shift_limit=30, val_shift_limit=20	1.0
Normalize	mean=(0.485, 0.456, 0.406), std=(0.229, 0.224, 0.225)	1.0

**Table 5:** Minimum FID (↓) values for all classes and models with corresponding MS-SSIM in parenthesis. Significant values in bold.

Model/Class	Viral	Healthy	Fungal	COVID-19	Bacterial	Mean
GAN	**91.33** (0.36)	**76.27** (0.35)	**95.43** (0.4)	**112.74** (0.38)	**101.86** (0.33)	**95.52**
DreamBooth	147.65 (0.41)	135.24 (0.48)	149.64 (0.41)	170.17 (0.48)	142.74 (0.48)	149.09
Fine-tuning	127.45 (0.41)	81.20 (0.37)	125.10 (0.41)	135.52 (0.37)	116.76 (0.36)	117.21
Unconditional	118.66 (0.67)	79.31 (0.67)	123.36 (0.39)	131.58 (0.38)	117.36 (0.34)	114.05
LoRA	166.86 (0.37)	124.18 (0.37)	163.68 (0.41)	177.75 (0.37)	144.11 (0.41)	155.32
mean	130.39	99.24	131.44	145.55	124.57	

**Table 6:** Medical assessment of quality and plausibility for all models with mean ± std over all classes.

Model	Quality	Plausibility
DreamBooth	1.64 ± 0.87	1.26 ± 0.54
Fine-tuning	2.94 ± 1.07	2.00 ± 0.81
GAN	**3.62** ± **1.06**	**2.25** ± **0.80**
LoRA	1.31 ± 0.65	1.06 ± 0.25
Unconditional	3.27 ± 1.01	2.02 ± 0.74

**Table 7:** Accuracy of classification models trained with additional synthetic images from the presented generative models. Between +100 and +10,000 images per class have been mixed into the training data. All results are reported as mean ± std of 5 independent training runs. Significant values are in bold.

Model/Architecture	ResNet50	EfficientNet-B0	EfficientNet-B1	ConvNeXt-T	ConvNeXt-S
Baseline	78.25 ± 1.14	78.71 ± 1.22	77.60 ± 1.19	78.25 ± 1.47	78.53 ± 0.37
**+100**					
DreamBooth	75.94 ± 1.44	79.54 ± 0.75	79.35 ± 1.50	78.06 ± 1.39	79.26 ± 1.77
Fine-tuning	76.22 ± 1.88	77.05 ± 1.76	77.33 ± 1.47	77.42 ± 1.57	80.37 ± 1.07
GAN	77.14 ± 2.19	77.97 ± 1.68	78.43 ± 1.28	78.99 ± 1.59	76.96 ± 2.00
LoRA	74.84 ± 1.56	76.13 ± 0.98	77.60 ± 0.63	78.43 ± 2.19	76.77 ± 1.71
Unconditional	75.67 ± 1.18	75.67 ± 0.84	78.53 ± 1.35	77.88 ± 1.30	79.45 ± 1.61
**+250**					
DreamBooth	78.25 ± 1.14	78.71 ± 2.41	**79.91 ± 1.81**	79.54 ± 1.66	78.62 ± 2.27
Fine-tuning	77.97 ± 1.11	76.96 ± 1.24	78.06 ± 1.64	76.68 ± 0.75	79.54 ± 1.56
GAN	76.31 ± 2.45	74.65 ± 1.34	76.87 ± 1.32	76.77 ± 1.59	78.16 ± 2.05
LoRA	73.82 ± 1.47	76.31 ± 1.39	76.87 ± 1.25	78.53 ± 1.03	79.08 ± 1.86
Unconditional	75.94 ± 1.22	76.22 ± 0.75	78.89 ± 1.28	79.17 ± 1.5	**81.11 ± 1.89**
**+500**					
DreamBooth	76.77 ± 0.80	79.54 ± 1.11	78.99 ± 2.11	78.43 ± 1.50	78.34 ± 2.47
Fine-tuning	75.02 ± 2.25	75.02 ± 2.32	77.51 ± 0.74	77.42 ± 0.77	77.05 ± 1.81
GAN	76.41 ± 1.28	74.29 ± 2.72	76.77 ± 1.71	77.60 ± 3.86	78.80 ± 1.05
LoRA	73.73 ± 1.65	76.59 ± 1.25	78.16 ± 1.26	78.34 ± 0.41	77.79 ± 1.88
Unconditional	78.16 ± 1.95	77.97 ± 0.79	78.53 ± 1.32	**79.82 ± 2.23**	80.28 ± 1.83
**+750**					
DreamBooth	78.25 ± 0.89	77.05 ± 1.11	79.72 ± 1.37	79.17 ± 2.53	78.89 ± 1.63
Fine-tuning	76.22 ± 1.35	77.51 ± 1.53	77.51 ± 0.98	76.87 ± 1.38	77.79 ± 1.83
GAN	75.58 ± 1.72	74.75 ± 2.27	75.58 ± 1.27	79.45 ± 1.92	77.70 ± 2.07
LoRA	74.65 ± 1.62	75.94 ± 2.13	76.68 ± 1.45	77.60 ± 1.47	78.06 ± 1.95
Unconditional	78.25 ± 1.32	77.24 ± 1.61	78.16 ± 2.66	78.34 ± 1.46	78.53 ± 2.31
**+1000**					
DreamBooth	**79.08 ± 1.39**	**80.46 ± 1.71**	79.82 ± 1.47	78.71 ± 2.30	79.26 ± 1.54
Fine-tuning	78.06 ± 0.95	75.39 ± 1.81	76.13 ± 1.68	76.96 ± 1.17	78.71 ± 0.84
GAN	76.59 ± 1.22	73.73 ± 1.51	75.02 ± 2.30	79.17 ± 1.35	78.06 ± 1.39
LoRA	76.31 ± 1.50	76.41 ± 0.98	77.51 ± 1.73	77.60 ± 1.97	78.06 ± 1.50
Unconditional	78.71 ± 1.22	75.85 ± 1.26	76.41 ± 2.21	78.34 ± 1.70	78.34 ± 1.67
**+5000**					
GAN	73.64 ± 1.25	75.21 ± 1.47	76.13 ± 1.18	76.13 ± 1.63	78.80 ± 1.54
**+10,000**					
GAN	74.47 ± 1.95	76.50 ± 1.05	75.30 ± 1.56	78.71 ± 0.98	79.72 ± 2.04

**Table 8:** Average change in accuracy in percentage points for all models from baseline over all additional image brackets. All results are reported as mean ± std of 5 independent training runs. Significant values are in bold.

Model/Architecture	ResNet50	EfficientNet-B0	EfficientNet-B1	ConvNeXt-T	ConvNeXt-S	Overall
DreamBooth	−0.59	**0.35**	**1.96**	**0.53**	0.34	**0.52**
Fine−tuning	−1.55	−2.32	−0.29	−1.18	0.16	−1.04
GAN	−2.52	−3.41	−1.3	−0.13	−0.22	−1.51
LoRA	−3.58	−2.43	−0.24	−0.15	−0.58	−1.40
Unconditional	−0.90	−2.12	0.50	0.46	**1.01**	−0.21
mean	−1.88	−2.09	0.02	−0.10	0.12	

**Table 9:** Precision, recall, and F1-Score for all generative models for each class. Baseline and oversampling models as a reference. Significant values for each class and metric in bold.

	Bacterial	COVID-19	Fungal	Healthy	Viral
**Precision**					
Baseline	0.3260 ± 0.0498	**0.8292 ± 0.0573**	0.4946 ± 0.0141	**0.9641 ± 0.0028**	0.1329 ± 0.1097
Oversampling	0.0000 ± 0.0000	0.6425 ± 0.0970	0.4073 ± 0.0987	0.9287 ± 0.0176	0.2697 ± 0.0461
DreamBooth	**0.4618 ± 0.0550**	0.7466 ± 0.0933	0.5085 ± 0.0592	0.9368 ± 0.0209	0.1826 ± 0.1581
Fine-tuning	0.2955 ± 0.1510	0.7164 ± 0.0696	0.6073 ± 0.0537	0.9503 ± 0.0221	**0.5112 ± 0.1291**
GAN	0.4569 ± 0.0595	0.8144 ± 0.1343	0.5506 ± 0.0414	0.9348 ± 0.0179	0.0848 ± 0.1291
LoRA	0.3778 ± 0.0697	0.7160 ± 0.1297	0.5669 ± 0.0936	0.9501 ± 0.0029	0.3738 ± 0.0928
Unconditional	0.4254 ± 0.1318	0.7038 ± 0.0405	**0.6390 ± 0.0673**	0.9391 ± 0.0135	0.4383 ± 0.1233
**Recall**					
Baseline	0.3474 ± 0.0976	0.7478 ± 0.0928	0.5517 ± 0.0899	0.9923 ± 0.0049	0.1000 ± 0.0848
Oversampling	0.0000 ± 0.0000	0.7913 ± 0.0577	0.4207 ± 0.2291	**0.9969 ± 0.0038**	0.3500 ± 0.1837
DreamBooth	0.3684 ± 0.1104	**0.8174 ± 0.0174**	0.5034 ± 0.1536	0.9954 ± 0.0038	0.1375 ± 0.1275
Fine-tuning	0.3474 ± 0.2297	0.7478 ± 0.0887	0.6276 ± 0.1142	0.9892 ± 0.0062	0.2375 ± 0.0468
GAN	**0.4000 ± 0.1511**	0.8000 ± 0.0443	0.5931 ± 0.2108	0.9877 ± 0.0038	0.0875 ± 0.1458
LoRA	0.3053 ± 0.0614	0.8087 ± 0.0976	0.4138 ± 0.1731	0.9954 ± 0.0038	**0.3625 ± 0.1075**
Unconditional	0.2842 ± 0.0714	0.7826 ± 0.1603	**0.6690 ± 0.0516**	0.9923 ± 0.0000	0.2625 ± 0.0729
**F1-Score**					
Baseline	0.3307 ± 0.0579	0.7793 ± 0.0296	0.5179 ± 0.0386	**0.9780 ± 0.0016**	0.1136 ± 0.0949
Oversampling	0.0000 ± 0.0000	0.7046 ± 0.0696	0.3958 ± 0.1615	0.9615 ± 0.0080	0.2868 ± 0.0864
DreamBooth	0.3943 ± 0.0486	0.7767 ± 0.0456	0.4990 ± 0.0983	0.9651 ± 0.0109	0.1452 ± 0.1224
Fine-tuning	0.3071 ± 0.1677	0.7252 ± 0.0324	0.6069 ± 0.0340	0.9692 ± 0.0112	0.3164 ± 0.0465
GAN	**0.4054 ± 0.0550**	**0.7983 ± 0.0536**	0.5507 ± 0.0959	0.9604 ± 0.0093	0.0854 ± 0.1368
LoRA	0.3272 ± 0.0304	0.7447 ± 0.0428	0.4613 ± 0.1171	0.9722 ± 0.0030	**0.3546 ± 0.0660**
Unconditional	0.3321 ± 0.0764	0.7325 ± 0.0906	**0.6497 ± 0.0341**	0.9649 ± 0.0071	0.3266 ± 0.0876

## Data Availability

The data that support the findings of this study are not openly available due to relevant data protection laws for human data. A sample of the data will be made available upon reasonable academic request from the corresponding author. The 70,000 synthetic images are available at https://huggingface.co/datasets/dschaudt42/synthetic_pneumonia (accessed on 30 November 2023).
